# MicroRNA-200a suppresses metastatic potential of side population cells in human hepatocellular carcinoma by decreasing ZEB2

**DOI:** 10.18632/oncotarget.3486

**Published:** 2015-03-08

**Authors:** Xisheng Yang, Jianlin Wang, Shibin Qu, Hongtao Zhang, Bai Ruan, Yuan Gao, Ben Ma, Xing Wang, Nan Wu, Xiaolei Li, Kefeng Dou, Haimin Li

**Affiliations:** ^1^ Department of Hepatobiliary Surgery, The Xijing Hospital of The Fourth Military Medical Uiversity, Xi'an, China

**Keywords:** MicroRNAs, Hepatocellular carcinoma, Side population, Metastasis

## Abstract

Although microRNA-200a (miR-200a) is frequently downregulated in cancer, its role in side population (SP) has not been investigated. In this study, 101 pairs of primary hepatocellular carcinoma (HCC) tissues and matched normal control tissues were analyzed for miR-200a expression and its clinicopathological value was determined. We found that miR-200a was downregulated in HCC/SP and this was associated metastasis. MiR-200a suppressed metastasis of SP cells. Overexpression of miR-200a in SP cells decreased metastasis-related markers and expression of ZEB2. The associations between miR-200a, SP cells and ZEB2 were validated in HCC. These findings reveal that miR-200a suppresses metastasis of SP cells by downregulating ZEB2.

## INTRODUCTION

Metastasis is a major cause of death in patients with hepatocellular carcinoma (HCC), although survival has improved due to advances in surgical techniques [1].

Cancer stem cells (CSCs) are characterized by their capacity for indefinite self-renew and by their relative quiescence [2, 3]. In several types of cancer, side populations (SPs) have been shown to be enriched for cells with CSC-like activity and a CSC phenotype [4]. Recent studies have suggested that SP cells in human pancreatic cancer are characteristically chemoresistant [5]. Furthermore, a recent study reported that SP cells possess the ability to self-renew, and to divide asymmetrically into SP and non-SP cells [6]. Other reports have indicated that SP cells may serve as an ideal model for stem cell for research [7].

MicroRNAs (miRNAs) are frequently dysregulated in cancer where they may behave as tumor suppressor genes [8, 9] or oncogenes [10]. Recently, it was reported that miR-200a plays a crucial role in the development of cancer through its regulation of epithelial to mesenchymal transition (EMT), cell migration, proliferation and metastasis [11, 12, 13]. In breast and ovarian cancer, the down-regulation of miR-200a plays an important role in cancer metastasis [14, 15, 16]. EMT is a process by which epithelial cells lose their cell-cell adhesion and gain invasive properties, which leads to the acquisition of mesenchymal stem cell characteristics [17, 18]. Previously, it was shown that mesenchymal to epithelial transition (MET) is promoted by repression of the zinc finger E-box-binding 1 (ZEB1) and ZEB2 expression [19, 20]. In our previous study, global miRNA expression profiles of SP cells in F344 rat HCC cells and fetal liver cells were screened through a microarray platform. We found that miR-200a was significantly downregulated in the SP cells of HCC compared with the fetal liver cells [21].

In this study, we show that low expression of miR-200a promotes human HCC SP cells to metastasize through the transactivation of ZEB2 expression, which results in the induction of EMT.

## RESULTS

### Downregulation of miR-200a is associated with metastasis of HCC and poor prognosis

**Figure 1:**
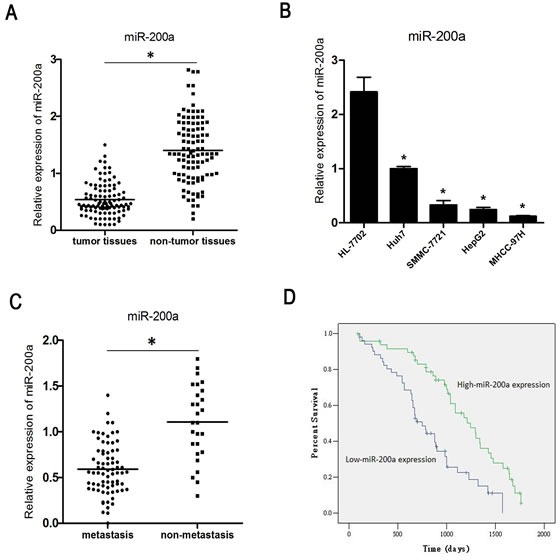
MiR-200a is down-regulated in HCC (A) The expression of miR-200a in HCC tissue specimens and in corresponding non-tumor tissues was measured by qRT-PCR. (B) The expression status of miR-200a in 4 human HCC cell lines and one normal human hepatocyte was measured by qRT-PCR. (C) The relative expression level of miR-200a in primary tumor samples with or without clinically confirmed metastasis was measured by qRT-PCR. (D) Kaplan-Meier analysis of the correlation between miR-200a expression and the overall survival of 101 patients with HCC. Patients in the low miR-200a expression group had a significantly shorter overall survival (*P* < 0.01); * *P* < 0.05, *t* test.

MiR-200a expression was analyzed in primary tumor specimens from 101 patients with HCC. The gene expression level of miR-200a was 0.59±0.08 in the tumor specimens and 1.40±0.41 in the corresponding non-tumor tissues (Fig. 1A). The index of miR-200a expression in the HCC cell lines MHCC-97H, HepG2, Huh-7 and SMMC-7721 was significantly lower than in the normal human hepatocyte cell line HL-7702 (Fig. 1B). We compared the low (51 cases) and high (50 cases) miR-200a expression groups (Table 1) and found that low expression of miR-200a strongly correlated with metastasis (*P*=0.011). The level of miR-200a expression was significantly lower in 74 cases of primary tumors with clinically confirmed metastasis compared with the 27 cases without metastasis (Fig. 1C).

The Kaplan-Meier analysis revealed that low miR-200a expression was associated with a shorter overall survival (*P* < 0.01) (Fig. 1D). The multivariate Cox regression analysis revealed that low expression of miR-200a (*P* < 0.001) as well as differentiation and metastasis were independent prognostic factors of patient survival (Table 2, lower panel).

**Table 1:** Association of miR-200a expression with the clinicopathologic factors of 101 patients with HCC

Characteristic	No of patients (*N* = 101)	miR-200a expression		*P* value
		High	low	
Gender				
Male	79	40(50.6%)	39(49.4%)	
Female	22	10(45.5%)	12(54.5%)	0.667
Age (years)				
≥50	68	33(48.5%)	35(51.5%)	
<50	33	17(51.5%)	16(48.5%)	0.778
Hepatitis B				
Positive (+)	71	32(45.1%)	39(54.9%)	
Negative (−)	30	18(60.0%)	12(40.0%)	0.170
Tumor size (cm)				
≥5	81	37(45.7%)	44(54.3%)	
<5	20	13(65.0%)	7(35.0%)	0.122
Metastasis				
Yes	74	31(41.2%)	43(58.8%)	
No	27	19(70.4%)	8(29.6%)	0.011*
Differentiation				
Moderately	69	33(47.8%)	36(52.2%)	
Poorly	32	17(53.1%)	15(46.9%)	0.620
AJCC stage				
0-II	68	36(52.9%)	32(47.1%)	
III-IV	33	14(42.4%)	19(57.6%)	0.321
AFP				
High	73	34(46.6%)	39(53.4%)	
Low	28	16(57.1%)	12(42.9%)	0.342

* Statistically significant difference; AJCC, American Joint Committee on Cancer; AFP, α-fetoprotein.

**Table 2:** Univariate and multivariate Cox regression analyses of overall survival in 101 patients with HCC

Molecular and Clinical Variables	Hazard Ratio (95% CI)	*P*-value
Univariate Analysis (Cox: Enter)		
MiR-200a	2.587 (1.577-4.425)	<0.001*
Gender	0.953 (0.559-1.626)	0.860
Age	1.161 (0.708-1.903)	0.555
Tumor size	1.051 (0.585-1.889)	0.869
Metastasis	2.843 (1.612-5.015)	<0.001*
Differentiation	0.587 (0.359-0.959)	0.033*
AFP	1.603 (0.959-2.679)	0.072
AJCC stage	0.590 (0.368-0.946)	0.028*
Multivariate Analysis (Cox: Forward LR)		
MiR-200a	2.483 (1.492-4.131)	<0.001*
Differentiation	0.541 (0.326-0.896)	0.017*
Metastasis	0.254 (1.421-4.540)	0.002*

* Statistically significant difference; Correlation of HCC patient overall survival with miR-200a expression level and clinical factors were analyzed by univariate (upper panel) and multivariate (below) Cox analysis.

### MiR-200a is inversely correlated with ZEB2 expression but is positively correlated with E-cadherin expression

**Figure 2:**
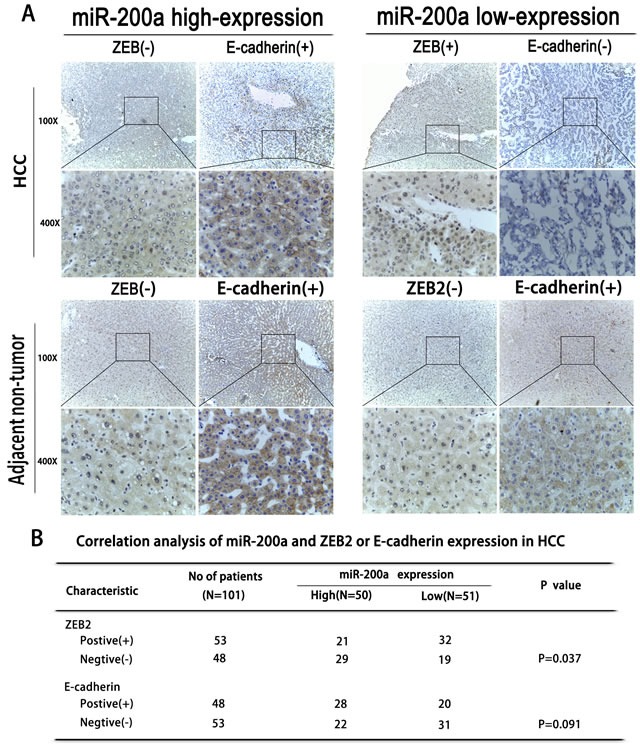
The correlation between miR-200a expression and EMT (A) An immunohistochemical analysis of miR-200a, ZEB2, and E-cadherin expression in HCC tissues and in adjacent non-tumor tissues. (B) The association between the expression of miR-200a and either ZEB2 or E-cadherin in human patients with HCC.

In our previous studies, we found that miR-200a inhibits the EMT process via the activation of transcriptional factors such as ZEB2, which is required during EMT; this has been thoroughly demonstrated in rat hepatic oval cells [22]. We further investigated the possible relationship between miR-200a, ZEB2 and E-cadherin expression in human HCC tissues. Immunohistochemistry revealed that miR-200a expression was inversely correlated with ZEB2 expression but was positively correlated with E-cadherin expression (Fig. 2A,B).

### Identification of side population

**Figure 3:**
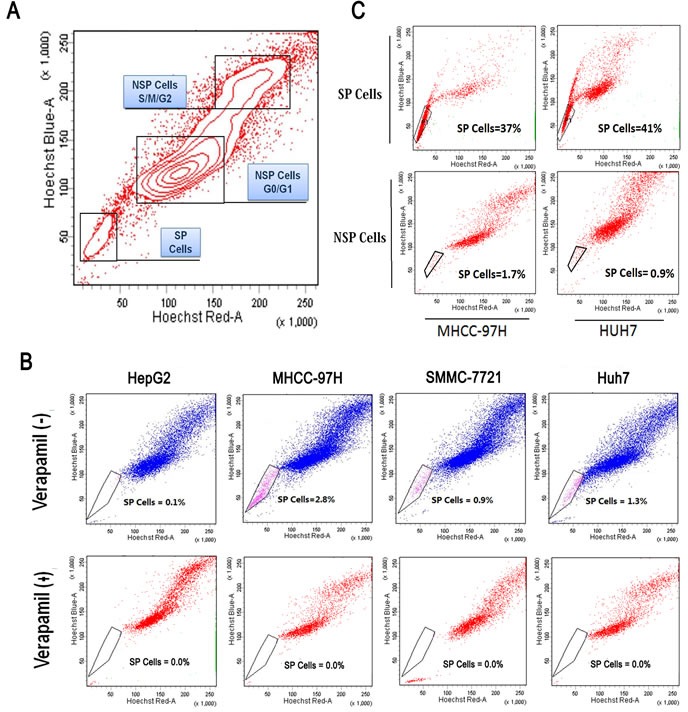
Identification of side population in HCC cell lines (A) A subpopulation was shown in a representative sample (red color-nuclear staining with Hoechst33342 dye; SP-side population; NSP-non side population). (B) SP cells of MHCC-97H, Huh7, SMMC-7721 and HepG2 were analyzed by dual wavelength FACS after incubation with Hoechst 33342. (C) A differentiation analysis of SP cells in a subpopulation of SP and NSP cells after four days in culture.

The subpopulation is shown in a representative sample (red color-nuclear staining with Hoechst33342 dye; SP-side population; NSP-non-side population). (Fig. 3A). HCC cells were analyzed by dual wavelength FACS after incubation with Hoechst 33342. Representative results that were analyzed by flow cytometry are shown (0.1% in HepG2, 2.8% in MHCC-97H, 0.9% in SMMC-7721, 1.3% in Huh7) (Fig. 3B). To understand the influence of differentiation in this subpopulation, SP cells were detected as a subpopulation of SP and NSP cells after 4 days in culture (37% and 1.7% in MHCC-97H, 41% and 0.9% in Huh7, respectively) (Fig. 3C).

### Characteristics of the subpopulation

**Figure 4:**
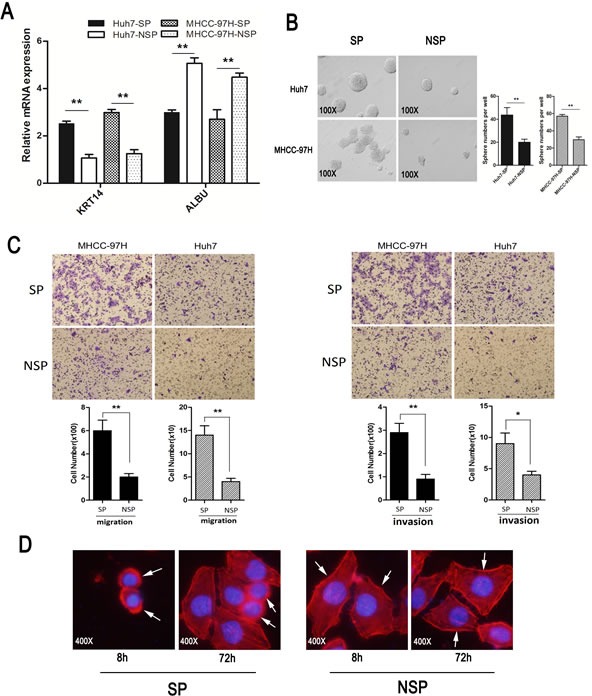
Characteristic of SP and NSP cells (A) The expression of ALBU (a marker of mature hepatocytes) and KRT14 (a marker of liver stem cells) in side population cells and in non-side population cells that were isolated from HCC cell lines. (B) Sphere formation assay: SP and non-SP cells were cultured in serum-free media for two weeks (magnification×100). (C) Cell invasion and migration assays: (Left panel, representative images of migration assay and quantification of cell number; Right panel, representative images of invasion assay and quantification of cell number). (D) Images of cells stained with Rhodamine Phalloidin and DAPI reflect the different morphologies (magnification×400). *p < 0.05, **p < 0.01, *t* test.

ALBU, a marker of mature hepatocytes, was lower in SP cells and higher in non-SP cells. Conversely, KRT14, a marker of liver stem cells, was highly expressed in SP cells but was weakly expressed in NSP cells (Fig. 4A). The number of spheres formed by SP cells from the Huh7 and MHCC-97H cell lines was significantly higher than the corresponding number of NSP cells. In addition, the SP cells were able to induce larger spheres than the non-SP cells (Fig. 4B).

The invasion and migration abilities of MHCC-97H and Huh7 cells were higher in SP cells than in NSP cells (Fig. 4C). The morphologic appearance of SP and NSP cells was determined after staining for F-actin filaments and after the nuclei were stained with Rhodamine Phalloidin and DAPI. Under fluorescence microscopy after different times in culture (either 8 h or 72 h after the subpopulation cells were sorted), the SP cells of the Huh7 cell line appeared confluent with little cytoplasm and large nucleolus, while the NSP cells demonstrated expanded cytoplasm and a considerably small pyknotic nucleolus (Fig. 4D).

### MiR-200a inhibits the metastasis of SP cells *in vitro*

**Figure 5:**
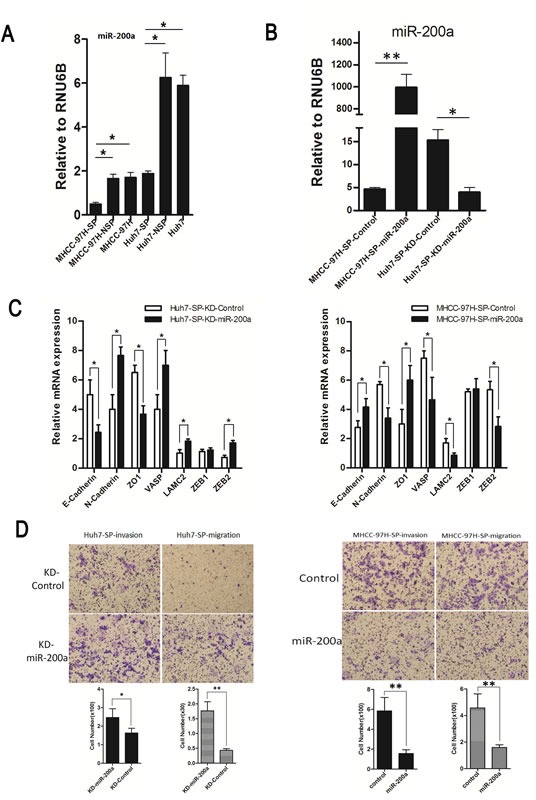
Functional analysis of miR-200a *in vitro* (A) MiR-200a expression in a subpopulation in human HCC cell lines. (B) Expression of miR-200a following transfection was confirmed by qRT-PCR. (C) Relative mRNA expression of metastasis-related markers in groups with and without miR-200a induction. (D) Cell invasion assay: (upper panel, representative images of invasion assay; bottom panel, quantification of cell number); Cell migration assay: (upper panel, representative images of migration assay; bottom panel, quantification of cell number). *p < 0.05, **p < 0.01, *t* test.

The index of miR-200a expression in SP cells of MHCC-97H and Huh7 was significantly lower than that in NSP cells from both the MHCC-97H and Huh7 cell lines (Fig. 5A). Expression of miR-200a after transfection was confirmed by qRT-PCR (Fig. 5B). Expression of metastasis-related markers was confirmed by qRT-PCR after transfection (Fig. 5C). E-cadherin and ZO-1 were over-expressed in the MHCC-97H-SP-miR-200a group but were weakly-expressed in the Huh7-SP-KD-miR-200a group. In contrast, the expression values of N-cadherin, VASP, LAMC2 and ZEB2 were opposite to those observed for the other above-mentioned genes. The upregulation of miR-200a expression decreased the ability of the MHCC-97H-SP cells to invade and migrate. Conversely, the inhibition of miR-200a expression in Huh7-SP cells enhanced the ability of the SP cells to invade and migrate (Fig. 5D).

### MiR-200a inhibits the metastasis of SP cells *in vivo*

**Figure 6:**
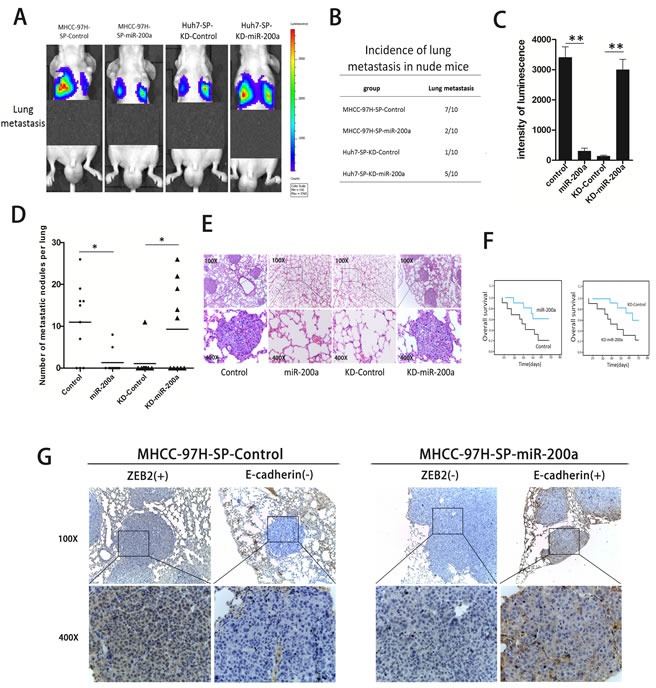
*In vivo* metastasis assays (A) Representative bioluminescent imaging (BLI) at 8 weeks is shown for the different groups. (B) The incidence of lung metastases in nude mice. (C) The intensity of luminescence in the different groups. (D) The number of metastatic nodules on the surface of the lungs in mice from the different groups. (E) Representative H&E staining of lung tissues is shown. (F) The overall survival of the nude mice. (G) An immunohistochemical analysis of miR-200a, ZEB2, and E-cadherin expression in metastatic nodules. *p < 0.05, **p < 0.01, *t* test.

SP cells were injected into the caudal vein of nude mice. Representative bioluminescent imaging (BLI) at 8 weeks is shown for the different groups (Fig. 6A). The *in vivo* metastatic assay showed that the upregulation of miR-200a decreased the incidence of lung metastasis and the number of metastatic lung nodules on the surface, but increased the overall survival time in the MHCC-97H-SP-miR-200a group. In contrast, the down-regulation of miR-200a increased the incidence of lung metastasis and the number of metastatic lung nodules on the surface but decreased the overall survival time in the Huh7-SP-KD-miR-200a group (Fig. 6B,C,D,F). Representative hematoxylin and eosin (H&E) staining showed that metastatic nodules were observed in the lungs of mice in the MHCC-97H-SP-Control group. There were no obvious metastatic nodules in the lungs of mice in the MHCC-97H-SP-miR-200a group. In contrast, metastatic nodules were observed in the lungs of mice in the Huh7-SP-KD-miR-200a group, whereas no metastatic nodules were found in mice in the Huh7-SP-KD-Control group (Fig. 6E). In addition, immunohistochemistry revealed that miR-200a expression was inversely correlated with ZEB2 expression but was positively correlated with E-cadherin expression (Fig. 6G).

### MiR-200a induces the metastasis of SP cells through the transactivation of ZEB2 expression

**Figure 7:**
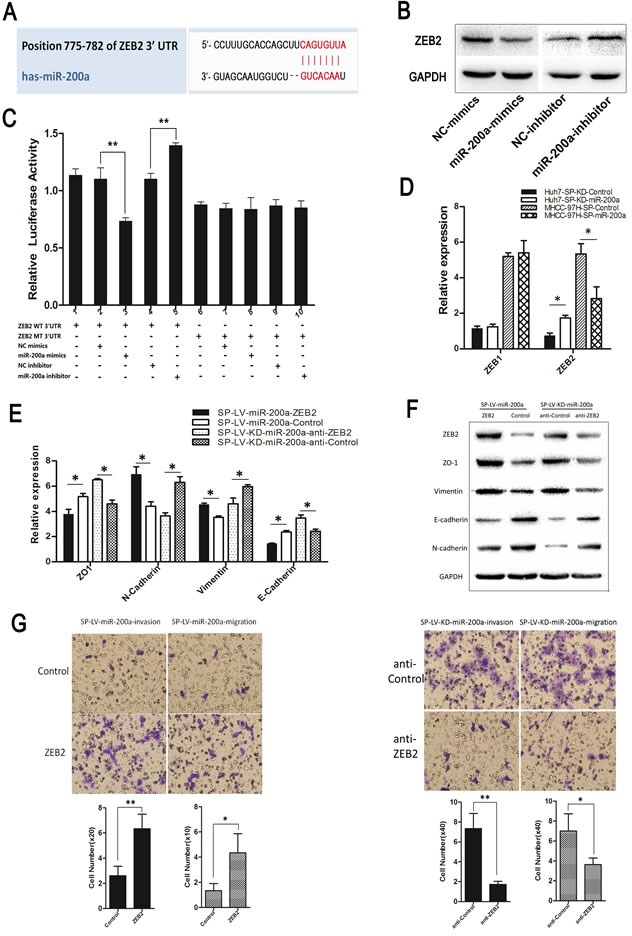
MiR-200a induces the metastasis of SP cells through the transactivation of ZEB2 expression (A) The miR-200a target site in the 3′UTR of ZEB2. (B) The up-regulation or down-regulation of miR-200a in MHCC-97H demonstrated an effect on ZEB2 according to western-blot. (C) Luciferase activity of ZEB2 after transfection with miR-200a mimics or an inhibitor. (D) Functional evaluation of miR-200a induction on its validated target, ZEB, in different groups by qRT-PCR. (E) Real-time PCR and (F) western-blot were used to detect the expression of EMT markers. (G) Following the infection of the MHCC-97H-SP-miR-200a cells and Huh7-SP-KD-miR-200a cells with ZEB2 siRNA and the designated SP-ZEB2 and SP-anti-ZEB2. The cell invasion and migration capacities were assessed with a transwell assay. *p < 0.05, **p < 0.01, *t* test.

The target site of miR-200a in the 3′UTR of ZEB2 was predicted (Fig. 7A). We also over-expressed or downregulated the miR-200a expression in MHCC-97H, and by western-blot we determined that miR-200a had the opposite function of ZEB2 (Fig. 7B). A luciferase reporter gene assay showed that, compared with the NC group, miR-200a mimics significantly inhibited the 3′UTR wild-type (WT) ZEB2 activity (*P* < 0.01) and a miR-200a inhibitor significantly increased its activity (*P* < 0.01). MiR-200a mimics and a miR-200a inhibitor had no effect on the activity of 3′UTR mutant (MT) ZEB2 (Fig. 7C) (Y-axis signifies the luciferase activity of ZEB2).

MiR-200a decreased ZEB2 and N-cadherin expression and increased E-cadherin and ZO1 expression in the MHCC-97H-SP-miR-200a group. The up-regulation of ZEB2 expression inhibited the increase in E-cadherin expression, ZO1 expression as well as the loss of N-cadherin expression induced by miR-200a. In contrast, the knockdown of miR-200a increased ZEB2 and N-cadherin expression and decreased E-cadherin and ZO1 expression in the Huh7-SP-KD-miR-200a group. The inhibition of ZEB2 dramatically attenuated the loss of E-cadherin expression, ZO1 expression and the increase in N-cadherin expression (Figs. 5C, 7D, E). Similar results were obtained in a western-blot analysis (Fig. 7F). The down-regulation of ZEB2 significantly reduced the migration and invasion abilities of the cells, which was induced by miR-200a knockdown. The up-regulation of ZEB2 rescued the miR-200a-induced decrease in cell migration and invasion (Fig. 7G).

## DISCUSSION

MiRNA expression profiling has shown that miR-200a is frequently downregulated [23, 24, 25, 26]. In this study we found that miR-200a expression was significantly downregulated in SP cells of human HCC cell lines and HCC tissues. As in the case with HCC cell lines, miR-200a expression was much lower in HCC tissues from patients who developed metastasis than in patients who did not develop metastasis. In addition, these clinical data strongly indicate that low expression of miR-200a contributes to the metastasis of HCC.

Recently, SP cells were isolated from the HeLa cell line and shared morphological characteristics with stem-like cells, including a rounder shape, smaller size and higher adherence, unlike the NSP cells [27]. In our research, ALBU was lower in SP cells but higher in NSP cells. Conversely, the expression of KRT14 was high in SP cells but was low in NSP cells. Additionally, SP cells had higher capacity for sphere formation and a greater capacity for invasion and migration than NSP cells. These results suggest that SP cells possess certain stem cell characteristics. In the last study, researchers primarily focused on functional studies of SP cells instead of on molecular mechanistic studies. In this study, we found that the down-regulation of miR-200a may promote the metastasis of SP cells, whereas the up-regulation of miR-200a inhibited the metastasis of SP cells.

EMT is driven by ZEB, SNAIL, AKT2 and Forkhead Box (FOX) transcription factors that activate genes associated with a mesenchymal phenotype and that repress epithelial marker genes [28, 29, 30, 31, 32]. These proteins bind to the promoter regions, which leads to the transcriptional inactivation of E-cadherin, ZO1 or the activation of N-cadherin and Vimentin [33, 34]. In this study, we found that miR-200a inhibited ZEB2 expression by binding to the ZEB2 promoter, which inhibits epithelial to mesenchymal transition. MiR-200a decreased N-cadherin expression and increased E-cadherin expression. The up-regulation of ZEB2 expression inhibited the increase in E-cadherin expression and the loss of N-cadherin expression. Thus, the miR-200a-mediated ZEB2/EMT signaling pathway is essential for SP cells in HCC cell lines to metastasize.

In conclusion, this study delineates the function of miR-200a in SP cells of HCC. Thus, further studies of SP cells from HCC and the components of this pathway may provide new insights into the treatment of this deadly disease.

## MATERIALS AND METHODS

### Flow cytometry

The cells were labeled with Hoechst 33342 (Sigma, USA) at a concentration of 5 ug/ml for SMMC-7721, Huh7 and 8 ug/ml for MHCC-97H. The cells were incubated for 90 minutes at 37°C in the absence or presence of 60 ug/ml verapamil (Sigma, USA). After staining, the cells were suspended in cold DMEM that contained 2 ug/ml propidium iodide (PI, Sigma, USA), 2% FBS and 10 mM HEPES, then passed through a 40 um mesh filter (BD, USA) and maintained at 4°C until flow cytometry analysis.

### *In vivo* metastasis assay

Male athymic nude mice (4-6 weeks old) were used. SP cells (1×104) were stably transfected with the luciferase gene, suspended in DMEM and injected into the caudal vein of nude mice. The location of tumors in the nude mice was detected by a bioluminescent imaging (BLI) system as previously described [35].

### Cell transfection

The human miR-200a gene was constructed in GV254 (Genechem, China) and designated as either LV-miR-200a or LV-knockdown-miR-200a (LV-KD-miR-200a). An empty vector was used as the negative control and was designated LV-control or LV-KD-control. ZEB2 siRNA and negative controls (NC) were obtained from Genechem Co. (China) and were designated SP-ZEB2 or SP-anti-ZEB2.

### Statistical analysis

Data were analyzed with SPSS 17.0 software. To evaluate significant differences between two matched paired groups or between two independent groups of samples, paired *t* tests and the Mann-Whitney *U* test were used, respectively. Survival curves were plotted according to the Kaplan-Meier method and were compared by the log-rank test. Data are expressed as the mean ± SD and *P* < 0.05 was considered statistically significant.
